# Artificial intelligence‐quantified schisis volume as a structural endpoint for gene therapy clinical trials in X‐linked retinoschisis

**DOI:** 10.1111/aos.17485

**Published:** 2025-03-29

**Authors:** Tien‐En Tan, Peilun Dai, Jonathan Hensman, Peter Kiraly, Beau J. Fenner, Yong Liu, Rick S. M. Goh, Ian C. Han, Daniel S. W. Ting, Camiel J. F. Boon, M. Dominik Fischer

**Affiliations:** ^1^ Singapore Eye Research Institute Singapore National Eye Centre Singapore Singapore; ^2^ Ophthalmology and Visual Sciences Academic Clinical Programme (EYE ACP), Duke‐NUS Medical School Singapore Singapore; ^3^ Oxford Eye Hospital Oxford University Hospitals NHS Foundation Trust Oxford UK; ^4^ Nuffield Laboratory of Ophthalmology, Nuffield Department of Clinical Neurosciences University of Oxford Oxford UK; ^5^ Institute of High Performance Computing Agency for Science, Technology and Research (A*STAR) Singapore Singapore; ^6^ Department of Ophthalmology Amsterdam University Medical Center Amsterdam The Netherlands; ^7^ The University of Iowa Institute for Vision Research The University of Iowa Iowa City Iowa USA; ^8^ Department of Ophthalmology and Visual Sciences, Carver College of Medicine The University of Iowa Iowa City Iowa USA; ^9^ Department of Ophthalmology Leiden University Medical Center Leiden The Netherlands; ^10^ Centre for Ophthalmology University Hospital Tuebingen Tuebingen Germany

**Keywords:** AI, clinical trial endpoints, gene therapy, inherited retinal disease, RS1, XLRS

## Abstract

**Purpose:**

To use artificial intelligence (AI) for quantifying schisis volume (ASV) in X‐linked retinoschisis (XLRS) for use as a structural endpoint in gene therapy clinical trials.

**Methods:**

We used data from Singapore, the United Kingdom, the Netherlands, and the United States. The AI model was developed on 250 optical coherence tomography (OCT) slices, with human annotation of schisis cavities (Dataset 1). ASV was quantified on Dataset 2 – 16 OCT scans from 8 eyes with XLRS at two time points, and Dataset 4 – 62 OCT scans from 31 eyes at two time points before and after carbonic anhydrase inhibitor (CAI) treatment. A clinical trial was simulated comparing CAI treatment against control. Changes in ASV, central subfield thickness (CST) and central foveal thickness (CFT) were compared. Effect size (Cohen's *d*) of the three structural endpoints was determined and used in sample size calculations for a future XLRS gene therapy clinical trial, at a 0.05 significance level and 80% power.

**Results:**

In the simulated clinical trial, all structural metrics showed greater reductions with intervention than with control, but only change in ASV reached statistical significance (*p* = 0.004). Cohen's *d* for ASV, CST and CFT were 0.972, 0.685 and 0.521, respectively. For the future gene therapy clinical trial, sample sizes required in each arm for ASV, CST and CFT were 18, 35 and 59 participants, respectively.

**Conclusions:**

ASV measurements can track changes in schisis volume in response to treatment. As an endpoint, ASV has a greater statistical effect size than CST/CFT, which reduces sample size requirements for future XLRS gene therapy clinical trials.

X‐linked retinoschisis (XLRS) is an inherited retinal disease (IRD) that affects about 1 in 5,000 to 1 in 20,000 individuals, and is one of the most important causes of juvenile macular degeneration in males (Hahn et al., 2022; Molday et al., 2012; Pimenides et al., 2005). Affected males typically present with bilateral reduction in visual acuity within the first two decades of life, and central vision loss is usually slowly progressive over time (Hahn et al., 2022). XLRS is caused by pathogenic variants in *RS1*, which encodes retinoschisin, an extracellular protein responsible for cellular adhesion between photoreceptors and bipolar cells, that is, therefore, important in maintaining the structural organization of the retina and signal transmission in the visual pathway (Hahn et al., 2022; Molday et al., 2012; Pennesi et al., 2018). Pathogenic variants in *RS1* result in characteristic abnormal splitting of the retinal layers, or retinoschisis, which occurs predominantly in the macula but can also be found in the retinal periphery in about 50% of cases (Hahn et al., 2022; Han et al., 2019; Hensman et al., 2024). Schisis cavities in the macula wax and wane over time, and eventually many patients develop macular atrophy (Fenner et al., 2023).

There is currently no disease‐modifying treatment available for XLRS. Treatment with oral or topical carbonic anhydrase inhibitors (CAIs) has been shown to reduce the volume of schisis cavities, but with only a modest effect on visual acuity, which may not persist over time (Ambrosio et al., 2021; Hensman et al., 2024). Gene therapy to express normal *RS1* and restore retinoschisin function could help to treat the underlying cause, potentially reducing schisis cavities and producing lasting improvements in vision (Cukras et al., 2018; Kjellstrom et al., 2007; Pennesi et al., 2022; Zeng et al., 2022). There is currently an ongoing phase 1/2 clinical trial investigating *RS1* gene therapy (ATSN‐201) with a spreading adeno‐associated viral (AAV) vector delivered by subretinal injection (NCT05878860).

Following the regulatory approval of voretigene neparvovec (Luxturna; Spark Therapeutics Inc.) in 2017, there has yet to be a second approved gene therapy for an IRD (Russell et al., 2017). This is in spite of the fact that there have been many gene therapy clinical trials conducted for IRDs. One significant challenge with clinical trials in gene therapy and other therapeutic strategies for IRDs is the availability of suitable and appropriate clinical trial endpoints (Christou et al., 2024; Igoe et al., 2024). Traditionally accepted regulatory endpoints such as a three‐line gain in best‐corrected visual acuity (BCVA) may be appropriate for diseases such as neovascular age‐related macular degeneration (AMD) or diabetic macular edema (DME), but are ill‐suited to clinical trials for IRDs (MacLaren et al., 2023). Considering that most IRDs cause progressive retinal degeneration, and that the mechanism of gene therapy is unlikely to cause reversal of established anatomic damage or photoreceptor loss, a more appropriate clinical trial endpoint for gene therapy might be halting or slowing the rate of disease progression, or maintenance of vision where the natural history in an untreated eye would be a decline. Quantitative biomarkers from imaging modalities such as optical coherence tomography (OCT) or fundus autofluorescence (FAF) imaging could be crucial to defining such endpoints. For example, in the OAKS and DERBY phase 3 registration clinical trials for pegcetacoplan treatment in dry AMD, the primary endpoint was the change from baseline in the area of geographic atrophy lesions quantified on FAF imaging (Heier et al., 2023). In OCT imaging, quantification of the ellipsoid zone (EZ), and the rate of change in EZ width/area over time has been proposed as an important structural biomarker and endpoint for retinitis pigmentosa (RP) (Christou et al., 2024; Igoe et al., 2024). Furthermore, when dealing with rare diseases that demonstrate slow disease progression, it is also crucial to have clinical trial endpoints that have high sensitivity to detect change, given sample size and feasibility constraints (Lambertus et al., 2017).

There is a clear need for the design and validation of better functional and structural endpoints in IRDs. In XLRS specifically, the characteristic schisis cavities that are a hallmark of the disease can be reliably imaged with macular OCT scans. Successful gene therapy should rescue the phenotype and result in the improvement or resolution of schisis cavities. Quantification of schisis cavity size and volume could be a key structural endpoint for clinical trials investigating therapies for XLRS. However, these schisis cavities are numerous and complex in shape and size, and are, therefore, difficult to manually quantify. Manual quantification of schisis cavities would be tedious, resource‐intensive, and would also need to demonstrate sufficient reliability. Various proxies have been used for examining changes in schisis cavities in XLRS, such as central subfield thickness (CST) and central foveal thickness (CFT), which are really measures of overall retinal thickening rather than direct measures of schisis volume. While relatively easy to quantify and measure, CST and CFT have thus far shown limited correlation with visual function in XLRS (Hahn et al., 2022; Hensman et al., 2024).

The primary aim of this study was to develop and validate an automated means of quantifying schisis volume in XLRS using artificial intelligence (AI). The secondary aims of this study were to explore structure–function correlation with AI‐quantified schisis volume (ASV) and to demonstrate the utility of ASV as a potential structural endpoint in future gene therapy clinical trials for the treatment of XLRS.

## 1 METHODS

This was an AI study using pooled data from multiple centres, including the Singapore National Eye Centre (SNEC; Singapore), the Oxford Eye Hospital (Oxford, United Kingdom), the Amsterdam University Medical Center (Amsterdam, The Netherlands), and the University of Iowa Institute for Vision Research (Iowa City, IA, USA). Ethics approval for this study was obtained from the respective ethics committees of each of these centres. Informed consent was obtained from all study participants, and this study adhered to the tenets of the Declaration of Helsinki.

### 1.1 Datasets

There were multiple non‐overlapping datasets used in this study (Table 1). Dataset 1 was the development dataset that was used for training and validation of the AI segmentation model. Dataset 1 consisted of 250 macular OCT slices, including 184 slices from 34 eyes with XLRS (all eyes had genotypically confirmed *RS1* variants apart from one, where the diagnosis of XLRS was made by typical findings accompanied by X‐linked inheritance pattern) and 66 slices from 11 normal eyes. In order to make the AI model as robust as possible, images that were included in this development dataset were selected to include a variety of pathological presentations, including eyes with large schisis cavities, minimal to no schisis, and late‐stage disease with retinal atrophy. All OCT slices in this dataset were manually annotated for schisis cavities by three graders (see details below).

**TABLE 1:** Description of datasets used in this study.

Dataset	Dataset 1	Dataset 2	Dataset 3	Dataset 4
Use of dataset	AI model development	AI model testing Control arm of simulated clinical trial	Reliability of ASV measurements with variation in OCT scan density	Structure–function correlation analysis Intervention arm of simulated clinical trial
Nature of dataset	Cross‐sectional (1 time‐point)	Longitudinal (2 time‐points)	Cross‐sectional (1 time‐point)	Longitudinal (2 time‐points)
Makeup of dataset	XLRS eyes Normal eyes	XLRS eyes (no treatment)	XLRS eyes	XLRS eyes (treated with CAIs)
Source	Amsterdam Oxford Singapore	Amsterdam Oxford	Amsterdam	Amsterdam Iowa
Size of dataset	250 OCT slices	16 OCT volume scans (8 eyes at 2 time‐points) 400 OCT slices	36 OCT volume scans (12 eyes, each with three scans taken in quick succession at 1 time‐point) 2052 OCT slices	62 OCT volume scans (31 eyes at 2 time‐points) 2354 OCT slices
Manual annotation of schisis cavities	Yes	Yes	No	No

Abbreviations: AI, artificial intelligence; ASV, AI‐quantified schisis volume; CAIs, carbonic anhydrase inhibitors; OCT, optical coherence tomography; XLRS, X‐linked retinoschisis.

Dataset 2 was the testing dataset that was used to evaluate the accuracy of the AI model schisis quantification. This dataset consisted of 16 whole volume macular OCT scans from 8 eyes with XLRS at two separate time‐points each, reflecting change in schisis volume over time without CAI treatment. The 8 OCT scans from the first time‐point were designated as Dataset 2.1, and the other 8 OCT scans from the second time‐point were designated as Dataset 2.2. There were 400 OCT slices in total, which were all manually annotated for schisis cavities by three graders (see details below). Dataset 2 was also used in the simulated clinical trial (see details below), as the control arm, reflecting natural history without CAI treatment.

Dataset 3 was used to determine the reliability of ASV measurements in macular OCT scans acquired with different scan densities and inter‐slice distances. This dataset consisted of 36 whole volume macular OCT scans from 12 eyes with XLRS, each with three macular OCT scans taken in quick succession at one time‐point. Each eye had three macular 20° × 20° OCT scans taken at the same visit, with varying inter‐slice distances of 60 μm (97 slices), 120 μm (49 slices), and 240 μm (25 slices). There were 2052 OCT slices in total, which were not manually annotated for schisis cavities.

Dataset 4 consisted of 62 whole volume macular OCT scans from 31 eyes with XLRS at two separate time‐points each, before and after treatment with oral or topical CAIs. This dataset was used for structure–function correlation analysis and was also used in the simulated clinical trial (see details below) as the intervention arm, reflecting change in schisis volume over time with CAI treatment. Fifty‐eight of these macular OCT scans (29 eyes) had accompanying best‐corrected visual acuity (BCVA) measurements, and 24 OCT scans (12 eyes) had accompanying microperimetry data (Macular Integrity Assessment [MAIA] Microperimeter; CenterVue). All OCT scans had CST and CFT values measured from Heidelberg Eye Explorer software (Heidelberg Engineering). CST was defined as the average retinal thickness in a 1 mm‐diameter circle centred on the fovea, and CFT was defined as the retinal thickness at a single point measured at the fovea. There were 2354 OCT slices in total, which were not manually annotated for schisis cavities.

### 1.2 Annotation of schisis cavities

In Datasets 1 and 2, all OCT slices were manually annotated for schisis cavities using an open‐source image analysis platform (3D Slicer; https://www.slicer.org/) (Fedorov et al., 2012). All the OCT slices were cropped, anonymized, and placed in random order for manual annotation by three graders (Grader 1 – JH, Grader 2 – PK, Grader 3– TET). Inter‐grader agreement was first established in a subset of 40 OCT slices from Dataset 1 that was mutually graded by all three graders, using Dice similarity coefficient analysis, where a value of 1 indicates perfect overlap between graders and 0 indicates no overlap at all. Thereafter, the remaining 210 slices from Dataset 1 and 400 slices from Dataset 2 were divided among the three graders for manual annotation.

### 1.3 Development of AI segmentation model

An AI image segmentation model was developed using U‐Net architecture with a ResNet34 backbone that was initialized with pre‐trained weights from the ImageNet database (Ronneberger et al., 2015). Dataset 1 was used as the development dataset and was split into training and validation components in a 9:1 ratio. Data partitioning was at the scan level, so that OCT slices from the same scan only appeared within the same partition of the dataset to prevent data leakage. OCT slices were pre‐processed by resizing and pixel value normalization. Model threshold was optimized based on the mean Dice coefficient on OCT slices where schisis was present.

### 1.4 Analyses performed

In Dataset 1, the subset of 40 OCT slices that were mutually graded by all three human graders was used to establish inter‐grader agreement in schisis annotation. Agreement was quantified by pairwise and three‐way Dice coefficients.

In Dataset 2, the accuracy of the AI model segmentation was tested at both the slice level and scan level. At the individual OCT slice level, the AI model segmentation was compared against human grader ground truth annotation and quantified by Dice coefficients. Annotation/segmentation of schisis cavities at the OCT slice level was converted to schisis volume measurements at the whole OCT scan level. Within each OCT slice, schisis area quantified in pixels was converted to mm^2^ by using scale bars. Schisis areas in each OCT slice were converted to schisis volume measurements in mm^3^ at the whole OCT scan level by using standardized inter‐slice distances, without interpolation. Each pixel on an OCT slice was assumed to occupy volume up to halfway between that slice and adjacent slices. In Datasets 2.1 and 2.2, at the whole OCT scan level, ASV values were compared against schisis volume based on human grader ground truth annotations (manual schisis volume [MSV]), and expressed as percentage AI estimation error: 100 × (ASV − MSV) / MSV. Longitudinal changes in schisis volume in the same eye across two time‐points were expressed as percentage changes for both ASV and MSV.

In Dataset 3, the AI segmentation model was used to quantify ASV at the whole scan level for all 36 OCT scans, without manual annotation. Mean ASV values for OCT scans obtained at 60, 120, and 240 μm inter‐slice distances were compared using a one‐way ANOVA test.

In Dataset 4, the AI segmentation model was used to quantify ASV at the whole scan level for all 62 OCT scans, without manual annotation. Mean ASV and BCVA values before and after treatment with CAIs were evaluated in 29 eyes (58 scans). Pearson correlation analysis was performed between ASV and BCVA across all 58 scans, and also by examining the longitudinal change in ASV and BCVA in the 29 eyes before and after CAI treatment. In the subset of data from the 24 scans (12 eyes) with accompanying microperimetry data, more structure–function correlation analysis was performed. Structural endpoints analysed were ASV, CST, and CFT. Functional endpoints analysed were BCVA, microperimetry mean macular sensitivity (MP MMS), and microperimetry mean volume sensitivity (MP MVS). Similarly, correlation analysis was performed between structural and functional endpoints across all 24 scans cross‐sectionally, and also by examining the longitudinal change in each of the endpoints across 12 eyes before and after CAI treatment.

In order to demonstrate the utility of ASV as a structural endpoint in XLRS, a clinical trial was simulated using Datasets 2 and 4. In this simulated clinical trial, eyes with XLRS were assigned to either of two treatment groups—the intervention arm where they received treatment with CAIs (Dataset 4, *n* = 31), and the control arm where they did not receive treatment (Dataset 2, *n* = 8). Both of these datasets had longitudinal data from eyes at two separate time points. Three potential structural endpoints were evaluated in this simulated clinical trial: mean change in ASV, mean change in CST, and mean change in CFT. The independent two‐sample *t*‐test was used to determine if there were significant differences between the two treatment groups using these three structural endpoints. The statistical effect size (Cohen's *d*) was determined for each of the structural endpoints and then used in sample size calculations for a future (hypothetical) gene therapy clinical trial for XLRS. In this hypothetical clinical trial, one arm would be assigned to a gene therapy intervention, and the other arm would be a control arm, allocated in a 1:1 ratio. For each of the three structural endpoints, we aimed to determine the sample size required in each arm, with a significance level of 0.05, to achieve power of 80% and 90%, respectively.

## 2 RESULTS

Inter‐grader agreement among the three human graders was established on a subset of 40 OCT slices from Dataset 1. Pairwise mean (SD) Dice coefficients between Grader 1–Grader 2, Grader 1–Grader 3 and Grader 2–Grader 3 were 0.857 (0.067), 0.872 (0.063) and 0.873 (0.061), respectively. Mean (SD) three‐way Dice coefficient among all three graders was 0.807 (0.086).

**FIGURE 1:**
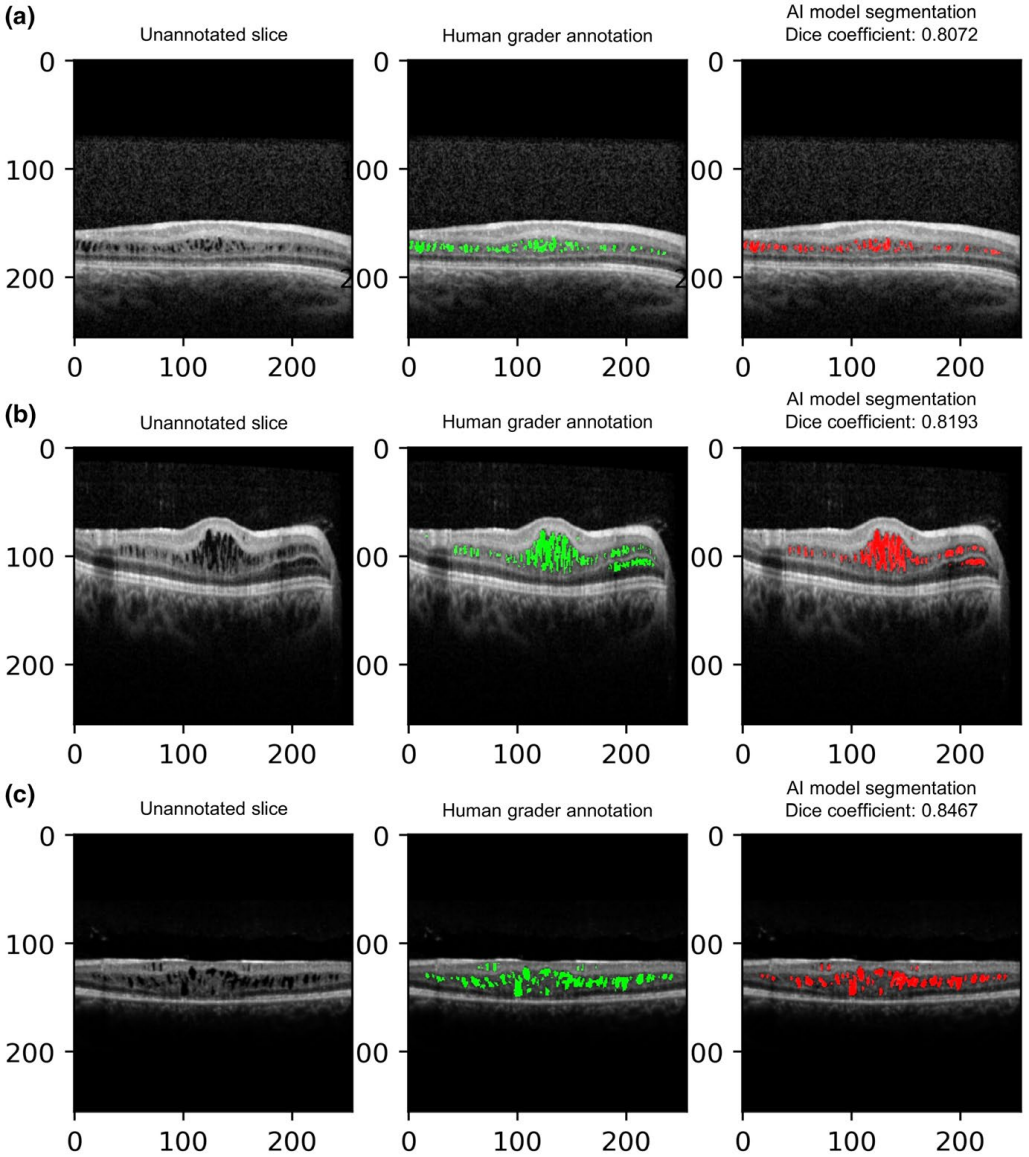
Examples from Dataset 2 of unannotated slices (left column), with corresponding human grader annotation (middle column) and AI model segmentation (right column), with Dice coefficients. (a) Small schisis cavities in the inner nuclear layer with good segmentation accuracy by the AI model. (b) Extensive schisis cavities in the inner nuclear and outer plexiform layers with vessel shadowing temporally mimicking schisis cavities in the outer nuclear layer. In this instance, the AI model correctly avoids segmented schisis areas in the area of shadowing in the outer nuclear layer, but does miss out on small areas in the outer plexiform layer nasally. (c) Schisis cavities in multiple layers including the ganglion cell layer, inner nuclear layer and outer nuclear layer. There is some outer retinal atrophy with a schisis cavity extending into the outer nuclear layer, that is accurately segmented by the AI model.

The AI segmentation model for schisis cavities was developed on Dataset 1 and tested on Dataset 2. The slice‐level median (IQR) Dice coefficient between the AI model segmentations and human grader ground truth annotations for Dataset 2 overall was 0.815 (0.680–0.898). The slice‐level median (IQR) Dice coefficients for Datasets 2.1 and 2.2 were 0.811 (0.704–0.890) and 0.817 (0.675–0.914), respectively. Figure 1 shows representative examples of unannotated OCT slices, along with the corresponding human grader annotation (ground truth) and AI model segmentations. Schisis segmentations/annotations were converted to schisis volume measurements at the whole scan level. Mean percentage AI estimation errors for Datasets 2.1 and 2.2 were −13.8% and −9.4%, respectively. When examining longitudinal change in schisis volume in the same eyes across two time‐points, mean percentage change in MSV was −33.8%, and mean percentage change in ASV was −29.0%.

Reliability of ASV measurements in macular OCT scans taken with varying scan density and inter‐slice distance was tested in Dataset 3. Mean (SD) ASV values for OCT scans with 60, 120, and 240 μm inter‐slice distances were 0.760 (0.531), 0.723 (0.533), and 0.794 (0.562) mm^3^, respectively. ASV values were not significantly different among the three groups (*p* = 0.950, one‐way ANOVA test).

In Dataset 4, with CAI treatment, mean (SD) ASV improved from 1.075 (1.084) mm^3^ to 0.484 (0.783) mm^3^, and mean (SD) BCVA improved from 59.4 (13.6) letters to 63.1 (15.5) letters. Correlation analysis cross‐sectionally across all 58 OCT scans showed no significant correlation between ASV and BCVA (*r* = −0.101, *p* = 0.452). When the data was examined longitudinally, in terms of the change in ASV and BCVA in 29 eyes after CAI treatment, there was a moderate correlation between change in ASV and change in BCVA (*r* = −0.493, *p* = 0.007).

**FIGURE 2:**
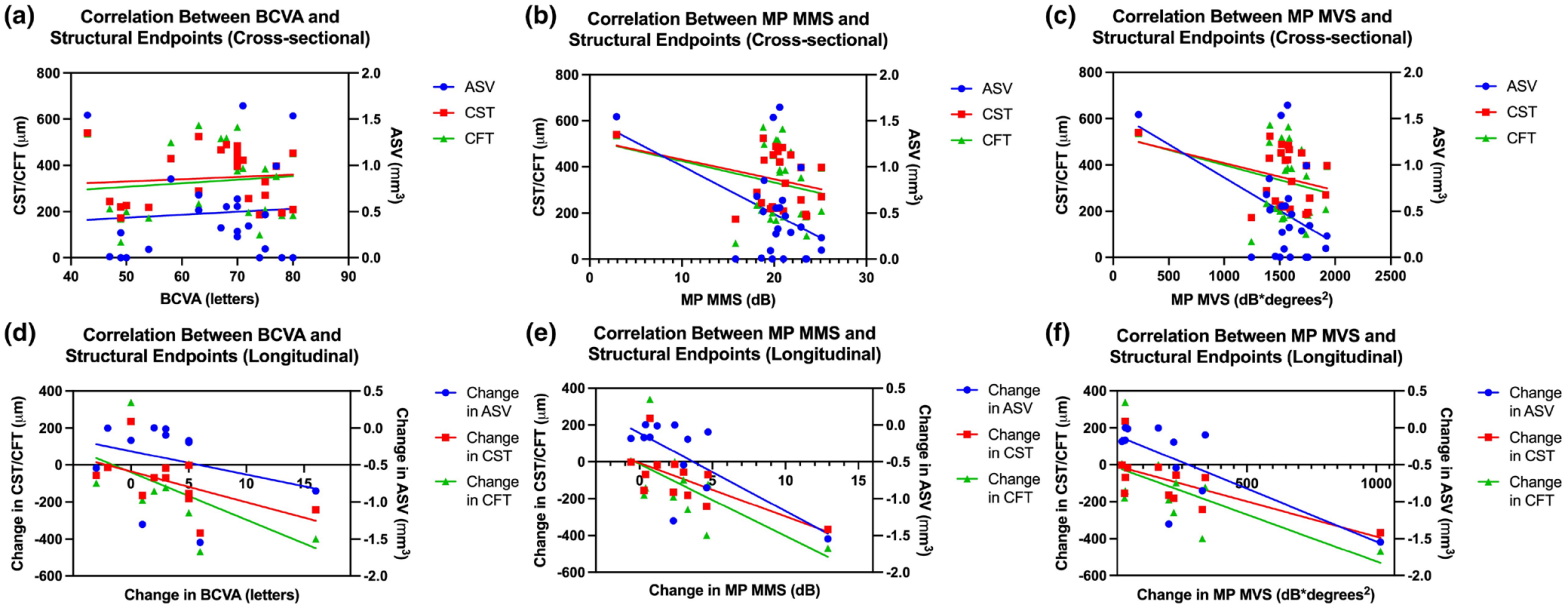
Structure–function correlation analysis in subset of eyes with microperimetry data. (a) Correlation between BCVA and different structural endpoints (*n* = 24). (b) Correlation between MP MMS and different structural endpoints (*n* = 24). (c) Correlation between MP MVS and different structural endpoints (*n* = 24). (d) Correlation between change in BCVA and change in different structural endpoints (*n* = 12). (e) Correlation between change in MP MMS and change in different structural endpoints (*n* = 12). (f) Correlation between change in MP MVS and change in different structural endpoints (*n* = 12).

More detailed structure–function correlation analysis was performed in the subset of eyes with microperimetry data. Similarly, this was performed cross‐sectionally across all 24 OCT scans (Table 2), as well as longitudinally, by looking at the correlation between change in structural endpoints and change in functional endpoints in the 12 eyes after CAI treatment (Table 3). Scatter plots of the correlation analyses performed are shown in Figure 2. Overall, microperimetry metrics (MP MMS and MP MVS) showed a stronger correlation with structural endpoints than BCVA. Specifically, in the cross‐sectional analysis, there was no significant correlation between BCVA and any of the structural endpoints. ASV showed moderate correlation with MP MMS (*r* = −0.436, *p* = 0.033) and MP MVS (*r* = −0.452, *p* = 0.027). CST and CFT showed a trend towards correlation with MP MMS and MP MVS, with *r* values ranging from −0.247 to −0.311, but these did not achieve statistical significance.

**TABLE 2:** Structure–function correlation analysis in a subset of eyes with microperimetry data—cross‐sectional (*n* = 24).

Pearson correlation coefficients	ASV	CST	CFT
BCVA	*r* = 0.072	*r* = 0.091	*r* = 0.109
	*p* = 0.738	*p* = 0.671	*p* = 0.612
MP MMS	***r* = −0.436**	*r* = −0.301	*r* = −0.247
	*p* = 0.033	*p* = 0.154	*p* = 0.244
MP MVS	***r* = −0.452**	*r* = −0.311	*r* = −0.260
	*p* = 0.027	*p* = 0.139	*p* = 0.219

*Note*: Values in bold indicate a significant correlation (*p* < 0.05).

Abbreviations: ASV, artificial intelligence‐quantified schisis volume; BCVA, best‐corrected visual acuity; CFT, central foveal thickness; CST, central subfield thickness; MP MMS, microperimetry mean macular sensitivity; MP MVS, microperimetry mean volume sensitivity.

**TABLE 3:** Structure–function correlation analysis in a subset of eyes with microperimetry data—longitudinal (*n* = 12).

Pearson correlation coefficients	Change in ASV	Change in CST	Change in CFT
Change in BCVA	*r* = −0.292	*r* = −0.544	***r* = −0.600**
	*p* = 0.357	*p* = 0.067	*p* = 0.039
Change in MP MMS	***r* = −0.710**	***r* = −0.692**	***r* = −0.665**
	*p* = 0.010	*p* = 0.013	*p* = 0.018
Change in MP MVS	***r* = −0.746**	***r* = −0.706**	***r* = −0.674**
	*p* = 0.005	*p* = 0.010	*p* = 0.016

*Note*: Values in bold indicate a significant correlation (*p* < 0.05).

Abbreviations: ASV, artificial intelligence‐quantified schisis volume; BCVA, best‐corrected visual acuity; CFT, central foveal thickness; CST, central subfield thickness; MP MMS, microperimetry mean macular sensitivity; MP MVS, microperimetry mean volume sensitivity.

Correlations were much stronger overall in the longitudinal structure–function analysis (Table 3). The strongest correlations were found between change in ASV and change in MP MMS (*r* = −0.710, *p* = 0.010), as well as change in MP MVS (*r* = −0.746, *p* = 0.005). Change in microperimetry metrics showed the strongest correlation with change in ASV, followed by change in CST and then change in CFT. On the other hand, change in BCVA showed the strongest correlation with change in CFT (*r* = −0.600, *p* = 0.039), followed by change in CST and then change in ASV.

In the simulated clinical trial of CAI treatment for XLRS using Datasets 2 (control arm) and 4 (intervention arm), all three structural metrics showed greater reductions in the intervention arm than the control arm (Table 4). However, only change in ASV showed a statistically significant difference (*p* = 0.004, independent two‐sample t test). Change in CST (*p* = 0.092, independent two‐sample *t* test) and change in CFT (*p* = 0.197, independent two‐sample *t* test) both failed to achieve statistical significance. Accordingly, change in ASV showed the largest effect size (Cohen's *d* = 0.972), while both change in CST (Cohen's *d* = 0.685) and change in CFT (Cohen's *d* = 0.521) showed moderate effect sizes.

**TABLE 4:** Evaluation of potential structural endpoints in a simulated clinical trial, using Dataset 2 as the control arm and Dataset 4 as the intervention arm.

Structural endpoint	Control arm (Dataset 2, *n* = 8)	Intervention arm (Dataset 4, *n* = 31)	*p*‐Value (independent two‐sample t test)	Cohen's *d*
Mean (SD) change in ASV (mm^3^)	−0.020 (0.351)	−0.568 (0.603)	0.004	0.972
Mean (SD) change in CST (μm)	−49.0 (93.5)	−155.7 (166.9)	0.092	0.685
Mean (SD) change in CFT (μm)	−81.6 (123.6)	−186.9 (216.3)	0.197	0.521

Abbreviations: ASV, AI‐quantified schisis volume; CFT, central foveal thickness; CST, central subfield thickness.

Sample size calculations were performed for a future (hypothetical) gene therapy clinical trial of XLRS, based on the effect sizes observed for each of the candidate structural endpoints (Table 5). Change in ASV required the lowest sample size, with 18 participants in each arm of the trial to achieve 80% power. In contrast, using change in CFT as the structural endpoint would require 59 participants in each arm of the trial to achieve the same level of statistical power.

**TABLE 5:** Sample sizes needed for different structural endpoints based on effect size and desired power.

	Sample size needed in each trial arm (*n*)		
	Change in ASV (Cohen's *d* = 0.972)	Change in CST (Cohen's *d* = 0.685)	Change in CFT (Cohen's *d* = 0.521)
80% power	18	35	59
90% power	24	46	79

Abbreviations: ASV, AI‐quantified schisis volume; CST, central subfield thickness; CFT, central foveal thickness.

## 3 DISCUSSION

In this study, we have developed and validated an AI segmentation model for automated quantification of schisis cavities in XLRS. This model is capable of quantification of schisis cavity area at the individual OCT slice level, as well as schisis cavity volume (ASV) at the whole volume OCT scan level, depending on the required use case. We have also demonstrated that ASV measurements at the OCT scan level are reliable even when applied to scans with varying inter‐slice distances and scan densities. Importantly, this AI model can be used to quantify and track longitudinal change in ASV over time in the same eye—this can be useful to track natural history, or as a structural endpoint in clinical trials to quantify and compare response to various interventions, such as treatment with gene therapy.

We have demonstrated the potential utility of this AI model and ASV as a structural endpoint for XLRS in two main ways. First, the AI model was used for automated quantification of ASV in a large dataset of 62 OCT volume scans with 2354 OCT slices from eyes with XLRS treated with CAIs (Dataset 4). Manual annotation of schisis cavities was not available in this dataset and would have required significant time and resource commitment. With the automated ASV measurements, we were able to track changes in ASV before and after CAI treatment and perform structure–function correlation analysis. We evaluated the relationship between BCVA and microperimetry metrics against three structural endpoints: ASV, CST, and CFT. In XLRS eyes treated with CAIs, we found that overall, microperimetry metrics (MP MMS, MP MVS) showed stronger correlation with structural endpoints than BCVA. Interestingly, for microperimetry metrics, the strongest structure–function correlation was found with ASV, followed by CST and then CFT. On the other hand, for BCVA, the strongest structure–function correlation was found with CFT, followed by CST and then ASV. This highlights some of the advantages and limitations of each of the structural endpoints evaluated. CFT was measured as retinal thickness at a single point at the fovea, and this would explain why it has the strongest correlation with BCVA, which typically is most closely related to foveal integrity. In contrast, CST represents retinal thickening over a 1 mm‐diameter zone around the fovea, and ASV measures schisis cavities that often extend much further out than just the central 1 mm zone. Similarly, BCVA only represents one aspect of central visual function, whereas microperimetry measures retinal sensitivity over a larger area of the macula (which in this study was a 10° diameter testing protocol), beyond just the fovea. Therefore, we would expect that structural endpoints that evaluate larger areas of the macula should be more strongly correlated with microperimetry metrics. BCVA is considered a suboptimal functional endpoint in most IRDs because it tends to be preserved until very late in the disease process, at which point it drops precipitously (Christou et al., 2024; Igoe et al., 2024). In contrast, microperimetry metrics, particularly volume sensitivity, may be more sensitive to disease progression and have therefore been proposed as important functional endpoints for gene therapy clinical trials in IRDs (Christou et al., 2024; Josan et al., 2021; Karuntu et al., 2024; MacLaren et al., 2023; Taylor et al., 2023). Between CST and ASV, again we would expect ASV to be more closely correlated with retinal function in XLRS because CST measures overall retinal thickening, which is a proxy for schisis cavities. CST would be subject to a floor effect and would also be affected by other factors that affect retinal thickness, such as atrophy. In contrast, ASV measures the specific pathological lesions of interest and should therefore have a better signal‐to‐noise ratio as a structural endpoint.

Second, in the simulated clinical trial, we demonstrated that ASV as a structural endpoint has the largest statistical effect size and the highest sensitivity to detect change when compared to CST and CFT. In comparing the two arms of the simulated clinical trial, CAI treatment resulted in larger reductions of schisis volume/retinal thickening compared to control—but only ASV was able to demonstrate statistically significant differences with the given sample size. Furthermore, when performing sample size calculations for a future gene therapy clinical trial for XLRS, the larger effect size of ASV translated to much smaller sample sizes required to achieve the desired statistical power. In our analyses, using ASV required about one‐third the sample size relative to CFT and about half the sample size relative to CST (Table 5). This would be advantageous in the conduct of any clinical trial, but particularly so in the field of rare diseases, such as XLRS and other IRDs. The fact that ASV can be easily quantified in an automated manner also makes it well suited for use in clinical trial settings, as it significantly reduces the need for resource‐intensive manual grading and annotation by a clinical trial reading centre.

Another group has previously reported developing an automated algorithm for quantifying cyst cavity volume (CCV) in XLRS on OCT scans (Pennesi et al., 2018). They first used a custom MATLAB software program to detect dark regions within the retina between the internal limiting membrane and retinal pigment epithelium, and then trained an AI neural network to classify the dark regions as schisis cavities or not. The algorithm they developed was used to quantify CCV in a cohort of 56 patients with XLRS and examine natural history over 18 months. They found no correlation between CCV and BCVA in their study (Pennesi et al., 2018). Information on the specific dataset and cohort used to develop this algorithm is limited. Our AI segmentation algorithm was developed on a multi‐centre cohort from four different countries, which intentionally included a wide range of pathologic presentations, including scans with late‐stage atrophic disease and large schisis cavities. We compared the ASV derived from the AI algorithm against other candidate structural endpoints such as CST and CFT and performed detailed structure–function correlation analysis with BCVA and microperimetry data. We also demonstrate the utility of ASV in terms of its large effect size and favourable sample size calculations for a future gene therapy clinical trial.

There are some limitations to our work that should be acknowledged. First, despite an overall acceptable level of agreement with the manual ground truth annotation (as quantified by Dice coefficients), some segmentation errors are inevitable on individual OCT slices. Our AI algorithm was designed to be somewhat conservative in identifying schisis cavities and tends to slightly underestimate schisis areas compared to human graders, as evidenced by the negative values of the percentage AI estimation errors on Datasets 2.1 and 2.2. However, if the AI model is being used to quantify and track longitudinal changes in ASV over time in the same eyes, this may not be as significant if the types of errors are consistent longitudinally (i.e. if the AI model reliably slightly underestimates schisis areas in a similar fashion across images in the same eye). In our dataset, the mean percentage changes in ASV (−29.0%) and MSV (−33.8%) across the two time‐points in Dataset 2 were quite similar. Having some segmentation errors in large datasets does not preclude the use of the AI algorithm in research trials or clinical practice. CST, which is widely used in both settings, is also an automated measurement provided by OCT viewing software and is frequently prone to segmentation or centration errors. It is standard protocol for clinical trial reading centres to manually check centration and segmentation before confirming CST values for analysis. Despite the need for some human quality control steps, the automated measurements do still reduce human resource requirements significantly and contribute crucial information for analysis. Second, standardized inter‐slice distances were used to derive both manual and AI‐derived volume measurements in this study. Actual inter‐slice distances vary slightly based on factors such as axial length and are available from the metadata of individual scans. Future work should include and use exact measured inter‐slice distances. Third, because of the retrospective nature of data collection in this study, scan acquisition parameters at the two‐time points for each eye were not standardized, and consecutive scans were not always registered. Horizontal and vertical fields of view for all scans in this study were consistent across different time‐points, but scan density/inter‐slice distances were not always the same. Nevertheless, we have shown in Dataset 3 that ASV measurements are reliable and not significantly affected by this factor. From our identification of some of these limitations, we recommend that for future gene therapy clinical trials in XLRS, scan acquisition parameters (field of view, inter‐slice distance) should be standardized and registered at all longitudinal time‐points.

In summary, we developed and validated an AI segmentation model for automated quantification of schisis area and volume in macular OCT scans of XLRS patients, which is capable of tracking longitudinal change over time and response to treatment. It is crucial that ASV, as an automated quantitative structural endpoint with superior signal‐to‐noise ratio, was shown to strongly correlate with important functional measures on microperimetry that are relevant for regulatory agencies. Perhaps most importantly, ASV measurements demonstrate a larger statistical effect size and greater sensitivity to detect change than CST and CFT measurements, which in turn translates to more favourable sample size requirements for future gene therapy clinical trials in XLRS.

## FUNDING INFORMATION

DSWT is supported by the National Medical Research Council (MOH‐000655‐00, MOH001014‐00), Duke‐NUS Medical School (Duke‐NUS/RSF/2021/0018, 05/FY2020/EX/15‐A58, 05/FY2022/EX/66‐A128), and the Agency for Science, Technology and Research (A20H4g2141, H20C6a0032). The sponsor or funding organization had no role in the design or conduct of this research.

## CONFLICT OF INTEREST STATEMENT

MDF is or has been (in the last 3 years) on the advisory board of and/or consulting and/or receiving honoraria/grant money/travel support from the following companies: Aavantgarde, Abbvie, Adelphi Values, Advent France Biotechnology, Adverum, Alder Therapeutics, Alphasights, Arctos Medical, Astellas, Atheneum, Atsena, Axiom Healthcare Strategies, Bayer, Biogen, BlueRock, Cambridge Consultants, Clarivate, Coave Therapeutics, Cogenita, Decision Resources, Dialectica, DORC, F‐Prime, Frontera Therapeutics, Guidepoint, Hoffmann Eitle, Ikarovec, iQUIVA, Janssen Research & Development, MedScape, Mogrify, Navigant, Novartis, NMD, PeerVoice, Physicians Education Resource, Revvity, Roche, RegenxBio, Sirion, Sovinnova Partners, Sparing Vision, SpliceBio, STZeyetrial, System Analytic, Techspert, TenPoint, Tern Therapeutics, THEA and Vindico Medical Education. He is Director of Fischer Consulting Limited and Oxford Medical Group Ltd. and holds a joint patent on the treatment of Retinitis Pigmentosa. He is an advisor to the University of Florida Bascom Palmer Institute and a Member of the scientific advisory board of the patient organizations Retina International and ProRetina. DSWT is a co‐inventor, with patents pending, for a deep learning system for diabetic retinopathy, glaucoma, and age‐related macular degeneration (SG Non‐Provisional Application number 10201706186V), and a computer‐implemented method for training an image classifier using weakly annotated training data (SG Provisional Patent Application number 10201901083Y), and is a co‐founder and shareholder of EyRIS, Singapore. No conflicting relationship exists for other authors.
